# Invited Commentary: Intraoperative Neural Monitoring for Early Vocal Cord Function Assessment After Thyroid Surgery—A Systematic Review and Meta-Analysis

**DOI:** 10.1007/s00268-021-06245-7

**Published:** 2021-08-19

**Authors:** Rick Schneider

**Affiliations:** Department of Visceral, Vascular and Endocrine Surgery, University Hospital Martin Luther University, Halle-Wittenberg Ernst-Grube-Straße 40, 06120 Halle (Saale), Germany

Kim et al. present a systematic review and meta-analysis of intraoperative nerve monitoring (IONM), based on a review of six major literature databases from their inception to March 2021, comparing IONM with laryngoscopic identification [1]. In contrast to the large number of previously published systematic reviews and meta-analyses of studies comparing IONM with visual nerve identification in the absence of IONM, with their heterogeneous and sometimes conflicting results, the authors in the present paper consider the diagnostic accuracy of intermittent and continuous neural monitoring in predicting vocal cord paralysis after thyroidectomy. Intraoperative neural monitoring is increasingly enjoying widespread use in cervical procedures, and especially after the technical improvements and facilitations in its application in recent years. This is absolutely conclusive since we know that an anatomically intact nerve does not allow prediction of postoperative function. Only intraoperative electrophysiologic intactness of nerve function can be considered as a surrogate parameter for intact postoperative vocal cord function [2].

Reviewing twenty-seven studies with 19,047 participants, Kim et al. found a diagnostic odds ratio for IONM of 152.96 with an area under the summary receiver operating characteristic curve of 0.966, indicating excellent diagnostic accuracy. The sensitivity, specificity, negative predictive value, and positive predictive value were 0.822, 0.978, 0.994, and 0.552, respectively. Considering only the accuracy of the IONM for temporary vocal cord paralysis, all presented values are even better. Subgroup analysis showed that the subgroups with intermediate (2011–2015) and later release (> 2015) had higher sensitivity than the early publication subgroup (< 2010). This discrepancy in results may be due to the standardization and quality control efforts of the IONM. Rigorous standardization and quality control, such as the International Standardization Guideline in 2011, [3] have managed to overcome the initial problems that plagued the IONM in the 1990s and 2000s. Together with standardization and technological advances, the sensitivity rating of IONM was over 90% after 2016, indicating the ability of IONM to be good enough to detect the postoperative vocal cord paralysis. To achieve these excellent results, the International Neural Monitoring Study Group’s L1, V1, R1, R2, V2, L2 approach and troubleshooting algorithm for loss of electromyographic signal must be followed [3]. For optimal IONM results, it is important to obtain nerve amplitudes ≥ 500 µV at baseline and use a stimulation current of 1 mA [4].

Based on this data analysis, it is recommended that IONM be routinely used as a component of intraoperative prediction of postoperative vocal cord function in thyroid procedures. Nonpredictable anatomic variants such as extralaryngeal nerve branching, Zuckerkandl’s tubercle, or a nonrecurrent nerve may also be present in noncomplex-appearing primary procedures for benign thyroid disease. In the case of loss of electromyographic signal on the first side in planned bilateral surgical procedures, IONM is also an integral part of the surgical concept of staged thyroidectomy [4]. If the electromyographic waveform of the first lobectomy site remains intact or shows recovery after temporary loss of electromyographic signal more than 50% of initial baseline in V2, the surgeon can safely proceed to the other lobe with the knowledge that nerve function of the ipsilateral side will be preserved after surgery, [4] with a specificity and negative predictive value of 99%.

A subgroup analysis of different forms of IONM showed that the diagnostic accuracy of continuous IONM was higher than that of intermittent IONM, implying a more diagnostically powerful tool. Because continuous IONM allows real-time acquisition of electrophysiological and neurophysiological signals through regular stimulation of the vagus nerve, it ensures a more accurate assessment of nerve integrity. Particularly since a large multicenter study by the International Neural Monitoring Study Group with 112 losses of electromyographic signal without intraoperative signal recovery demonstrated that 90% of all nerve lesions in anatomically intact nerves are traction-related, [5] only continuous IONM has the potential to identify precisely this mechanism of damage through typical combined electromyographic events so that it can be anticipated if detected early. As recently demonstrated in a large cohort study, continuous IONM is superior to intermittent IONM in preventing vocal cord paralysis. In more than 6000 patients with more than 12,000 nerves at risk, continuous IONM independently reduced early postoperative vocal cord paralysis 1.8-fold and permanent vocal cord paralysis 29.4-fold compared to intermittent IONM [6].

As Kim et al. concluded, the technology and knowledge of IONM have been accumulated and progressed over the past decades, and the predictive value of IONM in postoperative vocal cord paralysis has also improved. Moreover, the advances of continuous IONM technology could make a breakthrough in vocal cord evaluation after thyroid surgery. With the tremendous progress made in this dynamic field, CIONM has the potential to reflect the electrophysiological behavior of the nerve throughout the operation and thus not only attenuate or prevent loss of electrophysiologic signal but also make thyroidectomy transparent, also from a legal point of view.
